# Increased cell survival and cytogenetic integrity by spatial dose redistribution at a compact synchrotron X-ray source

**DOI:** 10.1371/journal.pone.0186005

**Published:** 2017-10-19

**Authors:** Karin Burger, Katarina Ilicic, Martin Dierolf, Benedikt Günther, Dietrich W. M. Walsh, Ernst Schmid, Elena Eggl, Klaus Achterhold, Bernhard Gleich, Stephanie E. Combs, Michael Molls, Thomas E. Schmid, Franz Pfeiffer, Jan J. Wilkens

**Affiliations:** 1 Department of Radiation Oncology, Klinikum rechts der Isar, Technical University of Munich, München, Germany; 2 Chair of Biomedical Physics, Department of Physics, Technical University of Munich, Garching, Germany; 3 Munich School of BioEngineering, Technical University of Munich, Garching, Germany; 4 Institute for Applied Physics and Metrology, Universität der Bundeswehr, Neubiberg, Germany; 5 Institute for Cell Biology, Ludwig-Maximilians Universität München, München, Germany; 6 Institute of Innovative Radiotherapy, Department of Radiation Sciences, Helmholtz Zentrum München, Oberschleißheim, Germany; 7 Department of Diagnostic and Interventional Radiology, Klinikum rechts der Isar, Technical University of Munich, München, Germany; 8 Institute for Advanced Study, Technical University of Munich, Garching, Germany; Northwestern University Feinberg School of Medicine, UNITED STATES

## Abstract

X-ray microbeam radiotherapy can potentially widen the therapeutic window due to a geometrical redistribution of the dose. However, high requirements on photon flux, beam collimation, and system stability restrict its application mainly to large-scale, cost-intensive synchrotron facilities. With a unique laser-based Compact Light Source using inverse Compton scattering, we investigated the translation of this promising radiotherapy technique to a machine of future clinical relevance. We performed in vitro colony-forming assays and chromosome aberration tests in normal tissue cells after microbeam irradiation compared to homogeneous irradiation at the same mean dose using 25 keV X-rays. The microplanar pattern was achieved with a tungsten slit array of 50 μm slit size and a spacing of 350 μm. Applying microbeams significantly increased cell survival for a mean dose above 2 Gy, which indicates fewer normal tissue complications. The observation of significantly less chromosome aberrations suggests a lower risk of second cancer development. Our findings provide valuable insight into the mechanisms of microbeam radiotherapy and prove its applicability at a compact synchrotron, which contributes to its future clinical translation.

## Introduction

X-ray microbeam radiation therapy (MRT) has shown high potential in terms of increased normal tissue tolerance and improved tumour control when compared to conventional radiotherapy. Undergoing a fast development in the last two decades, the idea of geometrical fractionation of the irradiation field was already implemented by Alban Köhler in 1909 using a mm-sized grid of iron wires for patient irradiations [1]. Reduced to the micrometer scale, many recent studies focus on the radiobiological effects of so-called *microbeams* with a beam width below 100 μm and a centre-to-centre spacing of 200-400 μm (e.g. [2–6]). Using such beams allows increasing the peak dose to several hundreds of Gray while maintaining a valley dose below the tolerance dose of normal tissue [7]. Therewith, the prescribed dose could even be given in a single treatment [2]. In vivo experiments performed in rats have demonstrated that MRT can prolong lifetime for radioresistant and aggressive brain tumours [4, 8]. In comparison to homogeneous irradiation fields, the concept of MRT allows for faster skin regeneration [9]. Furthermore, irradiation studies of duck embryos showed that immature, tumour-like vascular structure cannot repair the MRT damage as well as the mature, normal-tissue-like vascular structure [3, 6] resulting in higher tumour control. MRT studies in vitro and of excised tissue revealed differences in gene expression as radiation-induced immune modulations [10] and bystander effects caused at the tails of the planar microbeams [11, 12].

In contrast to conventional radiotherapy with MeV photons, keV-photons (∼ 100 keV mean energy) have to be used for MRT to maintain a collimated beam within the tissue and to keep the valley dose low. To avoid motion blurring, a high dose rate is required. These beam specifications are well met at large synchrotron facilities where most of the MRT research has been performed so far. Using the first commercially sold compact synchrotron X-ray source based on inverse Compton scattering, the Munich Compact Light Source (MuCLS), we investigate the translation of MRT to a laboratory-sized and more cost-efficient system that bridges the gap between conventional X-ray tubes and high-performance synchrotron facilities [13–15]. The MuCLS delivers quasi-monochromatic X-rays produced by inverse Compton scattering of low-energy laser photons by high-energetic electrons. With this, a lower electron energy compared to large-scale synchrotrons is sufficient to achieve keV X-rays. With a size of about 2 × 7m^2^ and lower operational costs, this system offers future clinical relevance. The X-ray energy can be tuned from 15 to 35 keV such that the MuCLS is well suited for preclinical experiments in vitro (cells and tissues) or in vivo (small animals). Here, we demonstrate that even with the current dose rate of up to 1 Gy/min MRT irradiations at the MuCLS yield promising results. Foreseen upgrades of the laser system of this prototype machine will reduce the irradiation time significantly by enhancing the flux [15]. Moreover, design adaptations on the electron injection and storage ring can easily enable energy upscaling to beyond 200 keV to reach a clinically relevant X-ray energy. We investigated three different cellular endpoints after MRT irradiation in comparison to homogeneously irradiated samples. To qualitatively verify the microbeam pattern, fluorescence images of DNA double-strand breaks were created by staining phosphorylated H2AX in HeLa cells. Secondly, we performed a clonogenic survival assay with CHO-K1 cells. Thirdly, we studied radiation-induced chromosomal aberrations in A_L_ cells to indirectly determine the cytogenetic damage caused by each irradiation geometry. We evaluated the amount of dicentric chromosomes (dicentrics) and centric rings as these chromosome aberration types are generally used for biological dosimetry after radiation exposure of humans. For both, the clonogenic cell assay and the chromosome aberration test, values of the relative biological effectiveness (RBE) were calculated at the MRT mean doses (averaged over irradiated area from peak-to-peak). In this study, the RBE is defined as the ratio of the dose in a homogeneous field to the MRT mean dose which yields the same biological effect (here: survival rate, dicentrics per cell, or centric rings per cell).

## Materials and methods

To investigate the effect of microbeam radiation therapy using a compact synchrotron based on inverse Compton scattering, three in vitro cell studies were carried out. We will briefly explain the production of X-rays and dose calculation at the MuCLS followed by a detailed description of cell preparation and handling and by the statistical analysis of the results.

### The Munich Compact Light Source

The Munich Compact Light Source (MuCLS) delivers quasi-monochromatic X-rays produced by inverse Compton scattering of low-energy laser photons at high-energetic electrons (Lyncean Technologies, Inc.). Electrons from a radiofrequency photocathode source achieve an energy of 25-44 MeV in a linear accelerator. The electron bunches are injected into a storage ring in which they circulate with a frequency of ∼ 65 MHz. An infrared laser (Nd-YAG) is enhanced by a high finesse bow-tie laser cavity, which shares one of the straight sections with the electron storage ring. The laser pulse repetition is matched to the electron bunch revolution to ensure collision with 65 MHz. At the intersection point, the beam waist of both bunches is focused down to about 50 × 50 μm^2^ defining the source size of the X-rays with an opening angle of the cone beam of 4 mrad. The X-ray energy *E* can be tuned via the electron energy from 15 to 35 keV with an intrinsic bandwidth of Δ*E*/*E*_*peak*_ = 3.0-4.3% (further machine specifications and beam characteristics can be found in ref. [15]). We chose the 25 keV configuration for our experiments yielding an average photon flux of up to 1 × 10^10^ ph/s to maximize the dose rate for cell material. Especially due to thermal drifts, fluctuations of the photon flux can occur. Hence, for correct dose deposition, the photon flux needs to be monitored permanently.

### Geometry of the cell irradiation setup

The cells were irradiated at about 1.7 m from the source with a beam size of ∼ 7 mm diameter. Spatially separated planar microbeams were created by a slit array made of a 200 μm thick tungsten foil with a slit size of 50 ± 3 μm and a tungsten bar size of ∼ 300 μm (Laser Micromachining Ltd.). We installed the slit array directly in front of the cells to avoid source blurring. This device was inserted for microbeam geometry and removed for homogeneous irradiations. The cells were seeded in a circle of either ∼ 3 cm (γ-H2AX assay) or about 4 mm diameter (clonogenic cell survival and chromosome aberration test) on a 6 μm Mylar foil, clamped between two stainless steel plates with a circular aperture in the irradiation field. Before irradiation, the cells were covered by a second Mylar foil and the culture medium was reduced to be available in horizontal position only (cell holder, cf. ref. [16]). For irradiation, the cell holder was placed vertically in the beam. Radiochromic films (Gafchromic EBT3, Ashland) directly attached to the cell holder allowed to verify the delivered dose for homogeneous irradiations. For live dose monitoring, a direct-detection single-photon counting detector (Pilatus 100K or Pilatus 200K, Dectris) with a 1 mm thick silicon sensor and a pixel size of 172 × 172 μm^2^ was placed at ∼ 16 m from the source. A radiographic image that shows the cell irradiation field illuminated through the tungsten slit array and details on the film evaluation are given in S1 Fig and as S1 Appendix in Supporting information respectively.

### Dose verification

The MuCLS spectrum of the 25 keV configuration was resolved with a Si-PIN detector (X-123, Amptek) and compared to Monte-Carlo simulations (Rod Loewen, personal communication, July 28, 2016). All energy-dependent variables included in the dose calculation were weighted with the simulated spectrum. During the experiment, the fluence, i.e. photons per pixel area, was recorded in a region of interest of the Pilatus detector, corrected for quantum efficiency. With inserted tungsten grating, the region of interest was adjusted to fit an integer number of periods of the peak-valley pattern. Next, the mean fluence at the Pilatus detector was computed from the respective region of interest. We obtained the mean fluence at the cells *ϕ*(*E*) by taking geometrical magnification and absorption of radiochromic film, Mylar windows, and air along the beampath into account. Therewith, we determined the absorbed dose to water via the energy-absorption coefficient $\mu_{\text{en},{\text{H}}_{2}\text{O}}$ and the density $\rho_{{\text{H}}_{\text{2}}\text{O}}$ considering the spectrum at the cells. Ignoring dose variations within the 10 μm thick cell layer, the dose *D* can be approximated by

$$\begin{array}{c} D=\frac{\int \phi \left(E\right)\cdot E\cdot \mu_{\text{en},H_{2}O}\left(E\right)\cdot dE}{\rho_{H_{2}O}}. \end{array}$$ (1)

Any absorption coefficients were retrieved from the NIST data base [17]. For the quantum efficiencies of both spectral and photon counting detector, the photoelectric absorption coefficient was used. To monitor flux variations and update the required irradiation time to achieve the desired mean dose, the fluence was recorded every second with the Pilatus detector. The dose rate varied from 0.01 to 1 Gy/min (with or without tungsten collimator). We treated sham-irradiated samples accordingly for several minutes or up to one hour. Systematic errors in dose calculation can arise from the determination of air absorption along the beampath, or the inaccuracy of absorption coefficients necessary for spectrum analysis and dose calculation via Eq (1) [18, 19]. Summarizing the sources of systematic errors using photon counting results in a dose uncertainty of ±10%. Additionally, statistical errors such as the choice of regions of interest on the photon counting detector and the manual timing of the X-ray shutter cannot be avoided but were minimized where possible. We determined statistical errors as sample standard deviation (SD) of the dose given to each replicate. As we compare homogeneous to MRT irradiation data based on the same dose calculation procedure, systematic errors in dose have no influence on the relative difference of the presented results.

### Cell preparation

#### γ-H2AX staining with HeLa cells

For the γ-H2AX assay, we irradiated HeLa cells in specifically designed cell containers [16] with a mean dose of 2 Gy. With an almost circular X-ray beam of about 7 mm diameter, only the center of the cell area (∼ 3 cm diameter) was irradiated. 30 min post-irradiation, the cells were fixed with 2% paraformaldehyde. Following the protocol described in ref. [20], the cells were permeabilized by three subsequent 5 min washing steps in phosphate-buffered saline (PBS) + 0.15% TritonX-100 (Sigma-Aldrich). Mouse anti-γ-H2AX antibody (Upstate) diluted 1:350 in PBS was added to the irradiated cells and left at 4°C overnight. Unbound antibody was removed by several washing and re-blocking steps before goat-F(ab’)2-anti-mouse antibody (Alexa488 labelled, 1:500, Invitrogen) was applied as secondary antibody. Finally, DNA was stained with DAPI (4’,6-diamidino-2-phenylindole) and a cover slip was mounted with a drop of Vectashield (Vector Laboratories) onto the cells. Microscopic γ-H2AX foci were immunolocalized and images were acquired using epifluorescence sectioning microscopy (Axiovert 200 M, Zeiss) via a LD A-Plan 20x/0.30 Ph 1 objective and a Zeiss AxioCamMRm resulting in a pixel size of 0.32 × 0.32 μm^2^.

#### Clonogenic cell survival with CHO-K1 cells

The colony-forming assay was performed using Chinese hamster ovary (CHO) cells. Monolayer cultures were grown in RPMI-1640 medium supplemented with 10% fetal calf serum, 100 units of penicillin, 100 μg of streptomycin per ml of culture medium, 2 mM L-glutamine, and 1 mM sodium pyruvate. Two weeks before the experiment, we thawed a frozen aliquot of cells. About four hours prior to irradiation, the cells were seeded on the Mylar foil pre-treated with Cell-TAK (Corning). The seeding area was restricted to a centred circle of about 4 mm to match the X-ray beam size [21]. The cell monolayers were incubated in a humidified atmosphere containing 5% CO_2_ and 95% air at 37°C. Immediately after irradiation, the cells were detached from the Mylar foil by trypsinization, counted, and reseeded into 12-well plates, where they were incubated for 5 days. Next, the colonies were fixed with methanol for 5 min and stained with 0.1% crystal violet for 2 min. Colonies, defined by at least 50 cells per colony, were counted automatically with a Bioreader (BIO-SYS GmbH) using identical settings for each experiment. Taking into account the number of seeded cells, the survival rate of the X-ray-irradiated samples was determined with respect to the plating efficiency of the sham-irradiated samples. Following the linear-quadratic regression model for the survival fraction *S* that depends on the dose *D* with *S* = exp(−(*αD* + *βD*^2^)), the corresponding sensitivity coefficients *α* and *β* were determined for the homogeneously irradiated cells as reference data and for the MRT treated cells (according to ref. [22]). The SciPy package for Python [23] was used for this non-linear least-square fitting of the data, which were weighted with the number of contributing replicates (3 to 5). From the resulting survival curve, we calculated the relative biological effectiveness (RBE).

#### Chromosome aberrations with A_L_ cells

We analysed radiation-induced chromosome aberrations in individual cells of a monolayer culture in the A_L_ cell line established for this purpose [24, 25]. These cells provide a standard set of CHO-K1 chromosomes with an additional single human chromosome 11. We applied the same growing procedure and handling before irradiation as described for the CHO-K1 cells. Immediately after irradiation, the cells were trypsinized and re-seeded in 4 ml RPMI-1640 medium supplemented with 10% fetal calf serum and antibiotics (penicillin/streptomycin) and incubated for 24 h at 37°C in a humidified atmosphere of 5% CO_2_ in air. Colcemid at a concentration of 0.03 μg/ml was added to the cultures 4 h after irradiation. Owing to the presence of colcemid, we synchronized the cycling A_L_ cells that were at the G1 phase of the cell cycle during irradiation [25]. The cultures were centrifuged, the supernatant removed and replaced by a hypotonic potassium chloride solution of 0.075 M at 37°C for 10 min. The cells were fixed with a 3:1 ratio of methanol to glacial acetic acid. Aliquots of fixative added dropwise to each sample were air dried, followed by cell staining with 2% of acetic Orcein for 10 min. Therewith, the cytoplasm of the cells was preserved and chromosome loss could be avoided. Chromosome preparation was performed according to the standardized laboratory procedure described by the IAEA for human lymphocytes [26, 27]. We estimated the radiation-induced cytogenetic damage via the yield of dicentrics and centric rings per cell. For each irradiation geometry, a non-linear least-square approximation was used to fit the following linear-quadratic model (implementation, cf. clonogenic cell survival) to the data for dicentrics and centric rings: dicentrics/cell = *αD* + *βD*^2^ + *c*. Due to the very low number of aberrations in the sham group of a total of 1700 scored cells, the offset value *c* to account for dicentrics in non-irradiated samples was set to zero, described as reasonable assumption in ref. [27].

### Statistical analysis of radiation-induced effects

For both, the survival fraction measured by the colony-forming assay and the cytogenetic damage resulting from the chromosome aberration test, the standard error of the mean (SEM) was calculated to account for the number of evaluated colonies and cells. Especially when determining the survival rate, different statistical errors due to cell handling can occur. To take this into account, we assumed the data to be normally distributed within a confidence interval of 68% yielding a *t*-factor (Student’s *t*-distribution) depending on the number of replicates with which the SEM was corrected (degrees of freedom = number of replicates −1). In contrast, the chromosome aberration test can be performed with very high accuracy so small dose fluctuations can be distinguished. Errors of fit parameters from the dose effect curves were propagated following Gaussian error propagation to determine the SEM of the RBE.

## Results

### Setup

**Fig 1:**
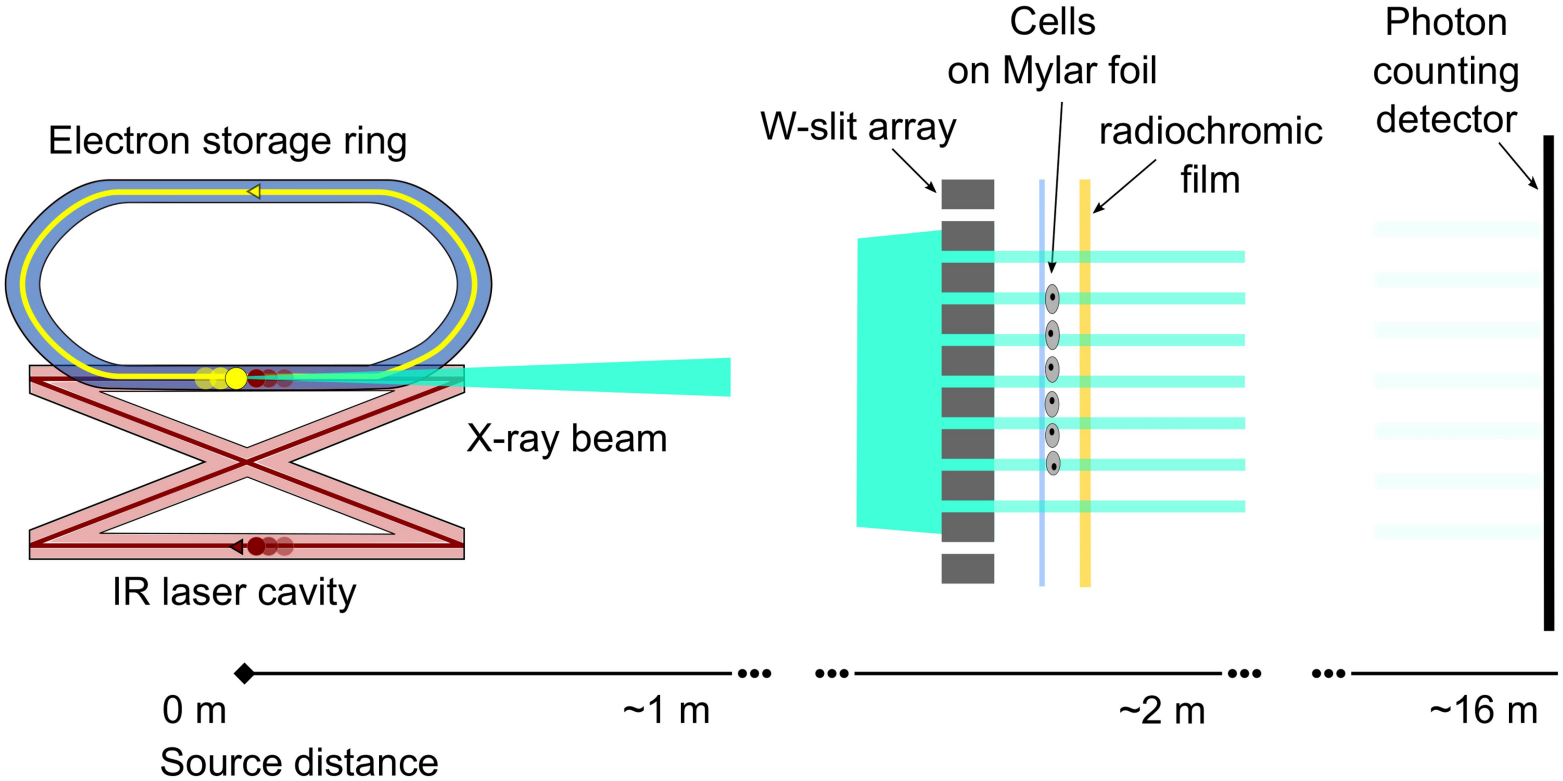
Schematic drawing of the microbeam setup at the Munich Compact Light Source (MuCLS). Accelerated electrons (yellow circles) are circulating in the storage ring at twice the rate as two laser pulses (red circles) in the laser cavity. Upon collision of the laser pulse with the electron bunch at the intersection point, a quasi-monochromatic X-ray beam (cyan) is produced. At a source-distance of ∼ 2 m, the tungsten slit array is positioned in the X-ray beam to create a microplanar radiation field. Directly behind the array, cells are situated in a dedicated cell holder. For dose verification, a radiochromic film is placed behind the cell holder. Live dose monitoring is performed with a photon counting detector at ∼ 16 m distance to the source. (Drawing not to scale).

Fig 1 shows a schematic representation of the cell irradiation setup at the MuCLS. The MuCLS is sketched comprising the electron storage ring with electrons (yellow) and the infrared laser cavity with photons (red) moving opposite to the electrons at the intersection point. Due to inverse Compton scattering, X-rays (cyan) are produced. The MuCLS was operated at 25 keV delivering a flux of up to 1 × 10^10^ ph/s. Microbeams were created with a 200 μm thick tungsten slit array with a slit size of 50 μm and 350 μm peak-to-peak distance. Directly behind the array, the cell holder was placed at a source distance of about 1.7 m. We attached a radiochromic film to verify the dose post-irradiation. Due to flux variations over time, transmitted photons were monitored with a photon counting detector and the photon number was corrected for all absorbing elements along the beam path. Therewith, the irradiation time was calculated to achieve the desired dose.

### γ-H2AX staining

**Fig 2:**
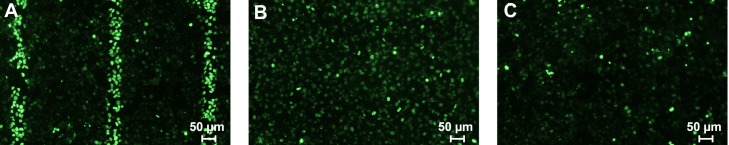
Fluorescence microscopy images using the γ-H2AX assay. DNA double-strand breaks in HeLa cells were stained after (A) microbeam irradiation and (B) homogeneous irradiation with a mean dose of 2 Gy, and (C) no irradiation. Equal acquisition, contrast, and scaling settings were applied.

To visualize the dose distribution in cells with the described setup, DNA double strand breaks were detected by antibodies binding to phosphorylated H2AX histones in HeLa cells. Green fluorescent dye (Alexa 488) was used to stain the sites of DNA double-strand breaks, shown in Fig 2 (compare with S2 Fig in Supporting Information for the position of nuclear DNA stained with DAPI). A mean dose of about 2 Gy was applied. The microbeam pattern with up to 14 Gy peak dose is well reflected via the DNA double strand breaks (cf. Fig 2(A)) and approximates the slit width and peak-to-peak distance given by the tungsten slit array. In Fig 2(B), the homogeneous irradiation of 2 Gy shows—as expected—an increase of the overall brightness with respect to the sham sample in Fig 2(C). Spontaneous or cell cycle dependent foci accumulation can appear in non-irradiated regions as background fluorescence visible in Fig 2(C).

### Clonogenic cell survival

**Fig 3:**
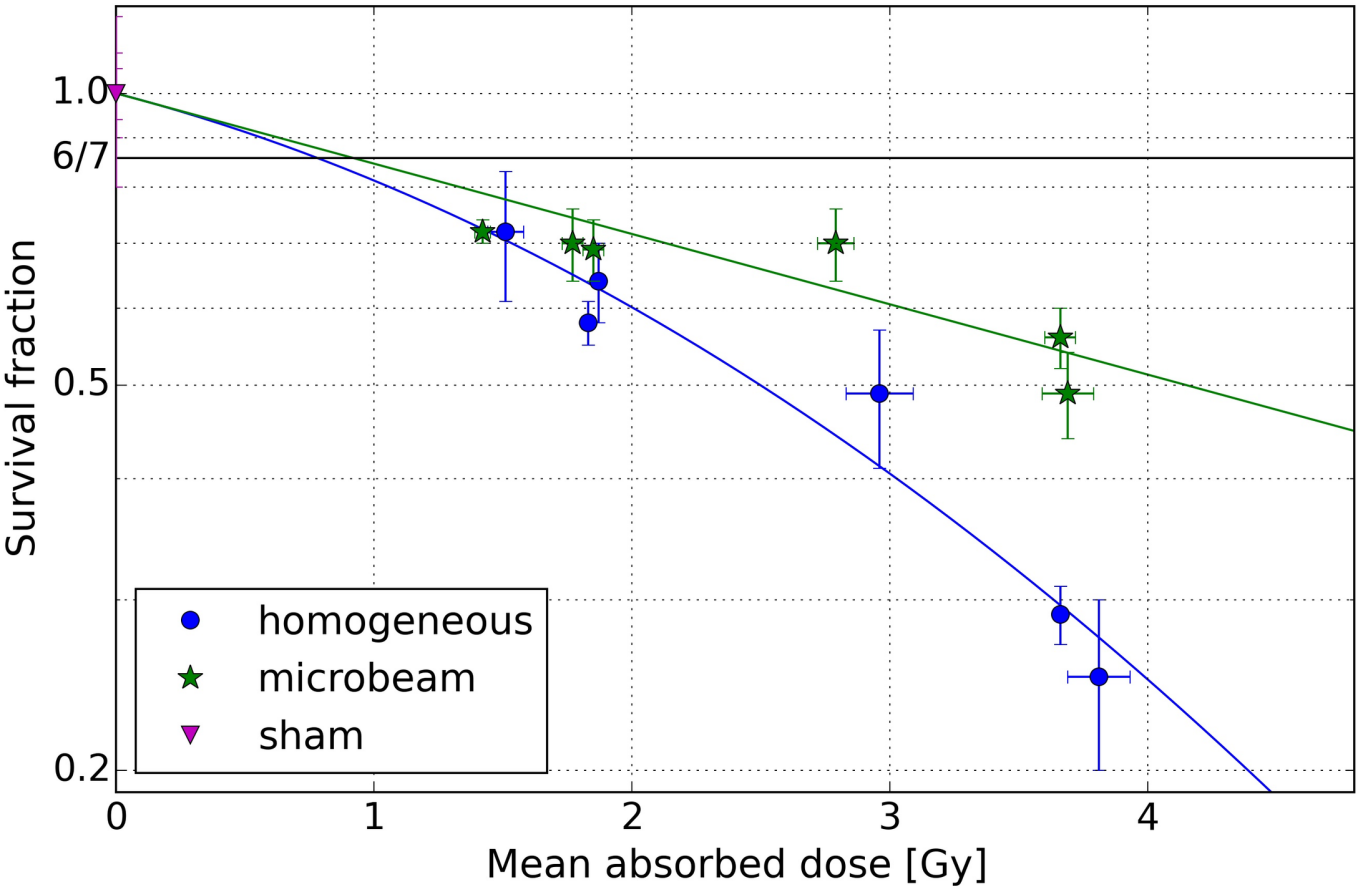
Dose-dependent survival fraction of CHO-K1 cells. Survival fraction (± SEM) of CHO-K1 cells plotted logarithmically with respect to the mean absorbed dose (± SD) determined by three independent clonogenic cell assays including three different doses each (0 Gy, < 2 Gy, > 2 Gy). Sham-irradiated cells are marked with magenta triangles, homogeneously irradiated cells with blue circles and microbeam treated cells by green stars. A linear-quadratic model was fitted to the survival data (blue and green solid lines) to estimate the relative biological effectiveness. The potential saturation of the survival fraction for microbeam irradiation was estimated by the geometry of the tungsten slit array with 6/7 (solid black line).

Cell survival was determined following microbeam and homogeneous beam irradiation of mean doses ranging from 1.4 to 3.7 Gy using a colony-forming assay with CHO-K1 cells in three independent experiments. For each experiment, a low dose (< 2 Gy), and a high dose (> 2 Gy) were delivered which resulted in six data points composed of 3 to 5 replicates to assess reproducibility and inter-test variability. In Fig 3, the survival fraction for both geometries (microbeam: green stars, homogeneous: blue circles), as well as sham-irradiated controls (magenta triangles) is shown for different absorbed doses. A linear-quadratic model was fitted to the survival data (blue and green solid lines). Additionally, a black solid line marks the survival rate of 6/7 for a lethal peak dose assuming a perfect tungsten slit array transferred into an idealized rectangular dose distribution within the cells yielding a survival rate of 1 (valley) or 0 (peak). Dose values in Fig 3 include statistical errors, represented as standard deviation (SD). The uncertainty on the survival data is given as standard error of the mean (SEM). Results from homogeneous irradiations were fitted with the sensitivity coefficients *α* = 0.160 ± 0.044 Gy^-1^ and *β* = 0.047 ± 0.015 Gy^-2^. As the survival of microbeam data does not show a quadratic dependence on the dose, only a linear coefficient *α* = 0.167 ± 0.066 Gy^-1^ was determined. Whereas at ∼ 1.4 Gy microbeam and homogeneously irradiated cells exhibit a similar survival fraction of ∼ 72%, the survival fractions following MRT decrease significantly slower above 1.9 Gy in contrast to the homogeneously irradiated cells (P value ranging from 0.01598 to 0.00007, two-tailed t-test for two independent means). Fitting a linear-quadratic model to the survival fractions suggests an even earlier divergence of the resulting dose effect curves at ∼ 0.5 Gy. The RBE reflects this behaviour as it decreases with increasing dose (cf. Table 1).

**Table 1:** Clonogenic cell survival—RBE values. Relative biological effectiveness (RBE) for equivalent cell survival of microbeam to homogeneous irradiations based on fitted data.

Exp. no.	Microb. dose [Gy]	RBE_survival_ (± SEM)
I	1.4	0.79 ± 0.17
I	2.8	0.67 ± 0.11
II	1.8	0.75 ± 0.14
II	3.7	0.62 ± 0.10
III	1.9	0.74 ± 0.14
III	3.7	0.62 ± 0.10

### Chromosome aberrations

**Fig 4:**
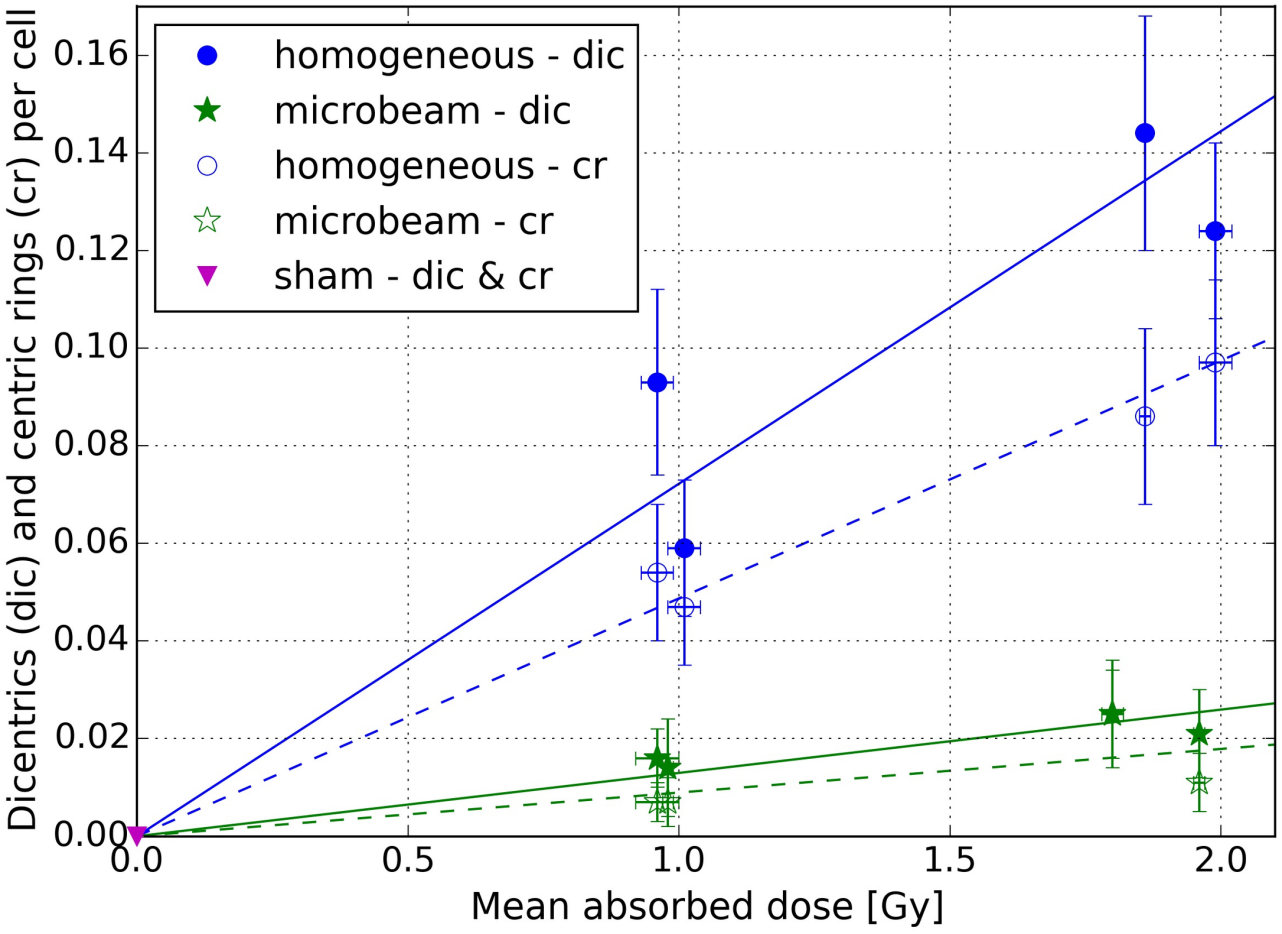
Dose-dependent chromosome aberrations in A_L_ cells. Dicentrics (dic) and centric rings (cr) per cell (± SEM) from three individual experiments for mean doses of 0, 1, 1.8, and 2 Gy. Homogeneous irradiation results are shown with blue circles, microbeam irradiation data with green stars and non-irradiated sham data with magenta triangles. Due to its small size, the error bar of the sham irradiation data is not visible at the given scale. A linear-quadratic model was fitted to the data (blue and green, solid and dashed lines). Solid symbols refer to dicentrics, open symbols to centric rings.

We studied dicentrics and centric rings in A_L_ cells following homogeneous, microbeam, and sham irradiation in three individual experiments (I, II, and III). Three replicates were analysed at each irradiation condition. A mean dose of 1 and 2 Gy was applied in the first experiment, 1 Gy in the second experiment and 1.8 Gy in the third experiment. This allowed us to improve statistical quality for the background aberrations and to investigate reproducibility. On average, 390 metaphase cells were scored per exposure condition for every experiment, limited by the irradiation field. Additionally, the intercellular distribution of dicentrics and centric rings for each experiment and irradiation setting was recorded (detailed data is given in S1 Table in Supporting information). To evaluate if the dicentric or centric ring yields follow a Poisson distribution, the dispersion ratio *σ*^2^/*μ* was calculated, with the standard error *σ* and the mean number of chromosome aberrations per cell *μ*. Its significance is stated by the *u*-value (cf. [28]) that has a standard normal distribution for a null hypothesis. For a 95% confidence interval, the standard deviation of the unit *u*-value is ±1.96. An obvious exception occurred for the 1 Gy microbeam case (experiment I), where only two dicentrics were found in two of all scored cells (294) causing an outlier with a *u*-value of 13.79. Otherwise, for dicentrics, the *u*-value ranges from -0.44 to -0.26, which suggests a trend towards underdispersion. In contrast, the *u*-value of the homogeneous data from -1.0 to 3.0 covers a larger interval slightly shifted towards overdispersion. Similarly, for the induction of centric rings, we measured underdispersion with a *u*-value of the microbeam data between -0.12 and -0.32, whereas the *u*-value of the homogeneous data ranges from -0.48 to 2.27 with a trend towards overdispersion. Dose-dependent dicentrics (dic) and centric rings (cr) per cell are presented in Fig 4, together with the respective least-square fits using a linear-quadratic regression model. As above, microbeam data are shown with green stars, homogeneous data with blue circles and non-irradiated data with magenta triangles. Solid symbols correspond to dicentrics, whereas open symbols represent centric ring data. Dose uncertainties are shown as SD referring to statistical errors. The uncertainty on the chromosome aberrations is given as standard error of the mean (SEM). For sham irradiation data, despite the different treatment times (see Materials and methods), the SEM is so small that the corresponding error bar is not visible at the given scale. From Fig 4, it becomes evident that the percentage of dicentrics and centric rings per cell increases similarly with the dose. As we chose a medium to high dose range of 1 to 2 Gy for the observation of chromosome aberrations at low keV energies (in chromosome aberration tests, doses typically range from 0.25 to 5 Gy with a maximum energy of several hundred keV [27]), the quadratic relation of the fit model described by *β* becomes negligible. The respective fit of homogeneous irradiation data yielded a linear coefficient *α*_dic_ in the dicentrics case of 0.072 ± 0.008 Gy^-1^ and for centric rings, *α*_cr_ approximated 0.049 ± 0.002 Gy^-1^. Microbeam data were fitted with *α*_dic_ = 0.015 ± 0.003 Gy^-1^ and *α*_cr_ = 0.008 ± 0.002 Gy^-1^. For both aberration types, dicentrics and centric rings per cell, we determined the RBE based on the fitted data. We observed a distinctly lower amount of dicentrics and centric rings per cell after MRT compared to homogeneous irradiation already at 1 Gy mean dose. For both, 1 and 2 Gy, the difference in cytogenetic damage is significant according to a two-tailed *t*-test for two independent means (dic: P value (1 Gy) = 0.00009, P value (2 Gy) < 0.00001; cr: P value (1 Gy) = 0.00044, P value (2 Gy) = 0.00004). For dicentrics, we estimated the same mean RBE_dic_ of 0.18 at 1 and ∼2 Gy absorbed dose (cf. Table 2). Equally, the mean RBE_cr_ from centric ring data accounts to 0.18 at 1 Gy and ∼ 2 Gy. The RBE values are given ± SEM, determined by the errors of the fit parameters.

**Table 2:** Chromosome aberrations—RBE values. Relative biological effectiveness (RBE) for equivalent dose effect (number of dicentrics (dic) or centric rings (cr) per cell) of microbeam to homogeneous irradiations based on fitted data.

Exp.no.	Microb. dose [Gy]	RBE_dic_ (± SEM)	RBE_cr_ (± SEM)
I	1.0	0.18 ± 0.01	0.18 ± 0.02
I	2.0	0.18 ± 0.01	0.18 ± 0.03
II	1.0	0.18 ± 0.01	0.18 ± 0.02
III	1.8	0.18 ± 0.01	0.18 ± 0.01

## Discussion

The results shown for microbeam and homogeneous irradiations strongly suggest that their radiobiological effect differs also at a quasi-monochromatic energy of 25 keV produced by a compact synchrotron source. Even if the peak doses are low with respect to several hundreds of Gray delivered in MRT studies at large synchrotron facilities, the sparing effect of MRT seems to hold true. The survival fractions measured with CHO-K1 cells prove that at least above a certain mean dose, less cells are lethally damaged after MRT irradiation than following homogeneous irradiation. In our study, this difference becomes significant above ∼ 2 Gy. Assuming a lethal peak dose and no valley dose in the geometry of the tungsten slit array, the non-irradiated area of 6/7 ≈ 85.7% should determine the minimum survival of the microbeam irradiated cells. For the maximum dose of 3.7 Gy, a survival lower than 60% was measured. The limit of 6/7 represents only a rough estimate of the survival fraction and disregards a variety of effects on the dose distribution. Geometrical effects such as source blurring (< 100 nm) and tungsten-slit-array imperfections deteriorate the sharpness of the microbeam edges as well as X-ray interactions with matter. The latter include, amongst others, scattering at tungsten edges, several μm range and distribution of secondary electrons in water (cell composition approximated with water, cf. [29]), strong contributions from the photoelectric effect at 25 keV that increases the valley dose [30], and bystander effects [12, 31]. However, the visualization of DNA double strand breaks via the γ-H2AX assay demonstrates that the applied microbeam pattern is transferred quite well into cell damage. Hence, the above-mentioned blurring effects should only be relevant in close vicinity to the microbeam. We did not observe an effect of sham irradiation time on the cell survival within the control group. Despite all these factors that could smoothen the microbeam dose distribution, a clearly superior cell survival after MRT is apparent above 2 Gy compared to homogeneous irradiations. Therefore, we can confirm a sparing effect using MRT. Consequently, using the same mean dose as in conventional radiotherapy, less damage in normal tissue, e.g. skin tissue, can be expected. Ibahim et al. [5] performed clonogenic cell assays for several cell lines after irradiation with ∼ 100 keV X-rays and calculated the biologically equivalent dose for the homogeneous case. For three different cell lines, a mean dose of 2.9, 3.1, and 3.2 Gy led to the same survival as a 50 Gy MRT peak dose with an estimated PVDR of 75 using a collimator with 7/8 of absorbing structure yielding a mean dose of 6.8 Gy. With our fitted RBE in this dose range, we would obtain an equivalent mean MRT dose of 5.20 to 5.94 Gy, which is quite close to the previously reported value. Considering the different energy settings, MRT pattern, and inter-cell-line variations, this comparison is not straightforward. However, it allows to conclude that using the MuCLS with lower energies but considerably higher peak-to-valley dose ratio, we can achieve a similar sparing effect as at large-scale synchrotron facilities (∼ 100 keV). For low-energy irradiation of superficial tumours, using the sparing effect of MRT for faster skin repair could be of high interest. In order to verify the benefits of this increased survival of normal tissue cells, in vivo studies are necessary. Moreover, for tumor treatment, the tumoricidal effect of MRT along with normal tissue sparing requires further investigation. 5-fold higher cytogenetic damage was observed using a conventional homogeneous beam geometry compared to MRT. The data from all three experiments demonstrate good reproducibility within the standard errors. With chromosome aberrations like dicentrics or centric rings, lethal damages were determined. In parallel, non-lethal aberrations, like translocations, occur that are more difficult to measure directly. With our approach, we can conclude indirectly on the relative amount of non-lethal radiation-induced cytogenetic damage that might cause second cancer development. Moreover, it has been demonstrated that inactivated dicentrics can lead to DNA damage and genomic instability [32]. Already at the lower dose of 1 Gy, MRT irradiated cells rarely showed dicentrics or centric rings compared to an 8-fold higher number after homogeneous irradiation. These two different chromosome aberrations led to almost equivalent RBE values, which highlights their correlation. From 1 to 2 Gy, the amount of cytogenetic damage in MRT irradiated cells stayed at a very low level in contrast to the much higher number of aberrations after homogeneous irradiation. Despite the high dose in the microbeam, the inter-cellular distribution of dicentrics or centric rings per cell did not change remarkably in contrast to observations made with other radiation qualities [25]. Moreover, the overall dispersion rate is even reduced, which indicates less severe cytogenetic damage compared to homogeneous data. Hence, our results suggest a lower second cancer risk following MRT irradiation than after homogeneous treatment. In conclusion, we could show that cytogenetic damage is reduced significantly using microbeams. This might be due to the lethal peak dose such that only at the microbeam edge, surviving cells are affected by X-ray radiation leading to a lower overall risk of chromosome aberrations.

## Conclusion

With the first microbeam radiation therapy experiments at a compact synchrotron X-ray source based on inverse Compton scattering, we demonstrated beneficial radiobiological effects in vitro in contrast to conventional, homogeneous irradiations. A slower decreasing cell survival with doses from 1 to 4 Gy resulting in a decreasing RBE value with dose suggests a tissue sparing effect for MRT irradiations. A very small amount of chromosome aberrations was observed at 1 and 2 Gy mean dose. The low RBE highlights the significantly reduced cytogenetic damage with respect to homogeneous irradiations at the same dose. This can be correlated to a lower risk of second cancer development. Following our promising results in vitro, studies in vivo including vascular structures are necessary to prove if we can achieve higher tumour control while sparing normal tissue by MRT with respect to homogeneous irradiations using a compact synchrotron X-ray source. Further development of CLS systems in terms of higher energies towards 200 keV, as well as flux, i.e. dose rate, is feasible which highlights their future clinical applicability.

## Supporting information

S1 Fig Radiographic image of the cell irradiation field. Radiographic image (taken with a Pilatus 200K detector) of the circular cell irradiation field (approx. 4 mm diameter) used to study clonogenic cell survival and chromosome aberrations. The tungsten slit array was inserted for microbeam creation. High intensity at the edges is an artifact of the deconvolution algorithm used to correct for source blurring.
(TIF)

S2 Fig Fluorescence microscopy images of DAPI-stained nuclear DNA in HeLa cells. To compare the cell nuclei distribution between the three γ-H2AX fluorescence microscopy images shown in the main manuscript, nuclear DNA was visualized via DAPI staining in the corresponding cell areas receiving (A) microbeam irradiation and (B) homogeneous irradiation with a mean dose of 2 Gy and (C) no irradiation. Equal acquisition, contrast, and scaling settings were applied. Slight differences in sharpness can be due to the mounting process of the Mylar foil with the cover slide. The dense cell distribution in (A) enables easier visualization of the grating structure in the γ-H2AX channel. Even though the distribution of the cells is more even in the homogeneous case compared to the sham (cf. (B) and (C), respectively), the increased brightness seen in the γ-H2AX channel for homogeneous irradiation is not related to a denser cell distribution.
(TIF)

S1 Appendix Radiochromic film verification. (PDF)

S1 Table Detailed data on chromosome aberrations. Frequency of dicentrics or centric rings per analyzed cell and their intercellular distribution in A_L_ cells after homogeneous and microbeam irradiation with 25 keV X-rays in three experiments (Exp. I, II, III). Three replicates were performed with each irradiation condition.
(PDF)
