# Supplementary figures and images for: Increased cell survival and cytogenetic integrity by spatial dose redistribution at a compact synchrotron X-ray source
Source: PLoS One. 2017 Oct 19;12(10):e0186005. doi: 10.1371/journal.pone.0186005 (PMC5648152; doi:10.1371/journal.pone.0186005)

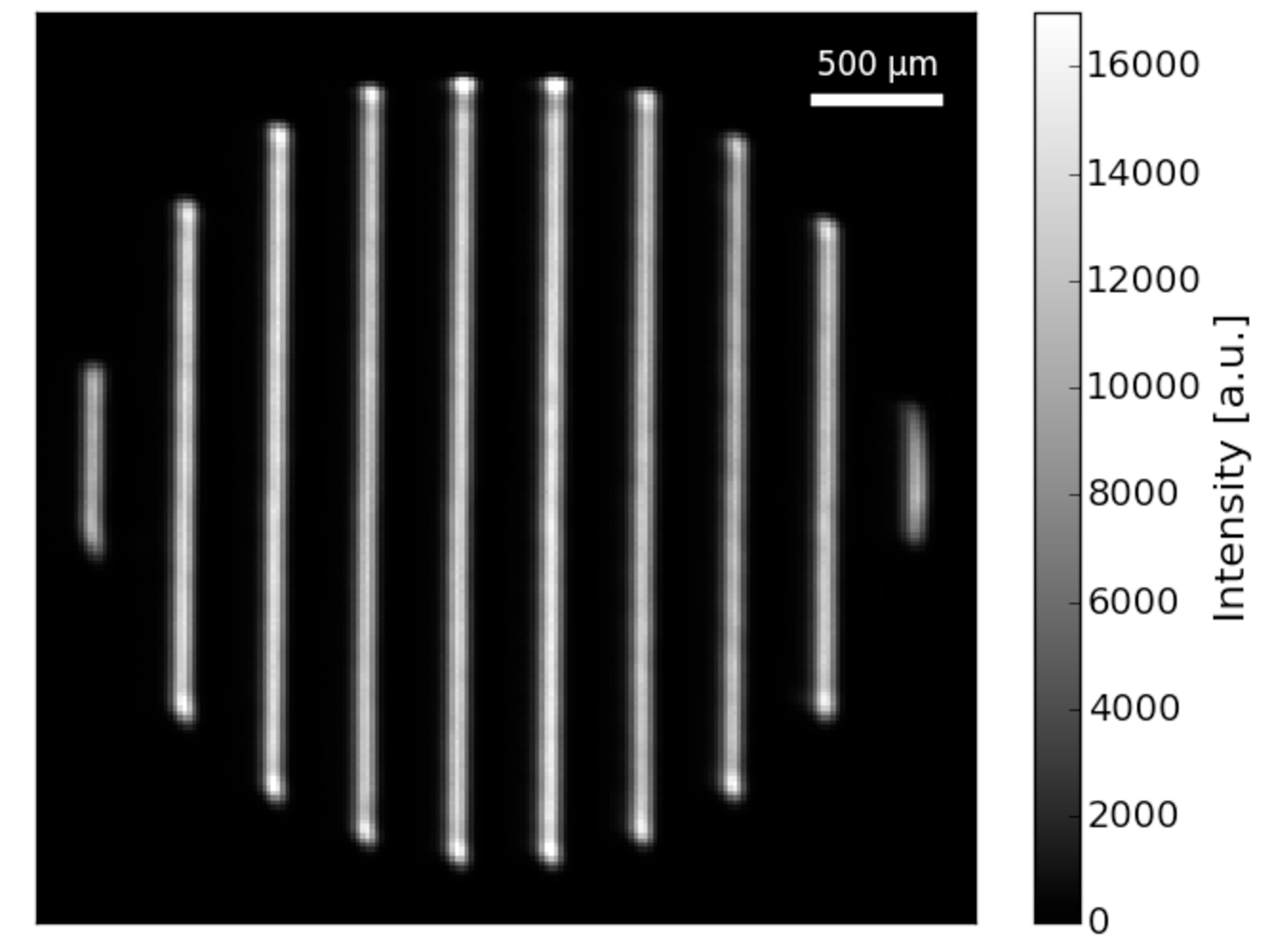

Supplement: S1 Fig — Radiographic image (taken with a Pilatus 200K detector) of the circular cell irradiation field (approx. 4 mm diameter) used to study clonogenic cell survival and chromosome aberrations. The tungsten slit array was inserted for microbeam creation. High intensity at the edges is an artifact of the deconvolution algorithm used to correct for source blurring. (TIF) [file pone.0186005.s001.tif]

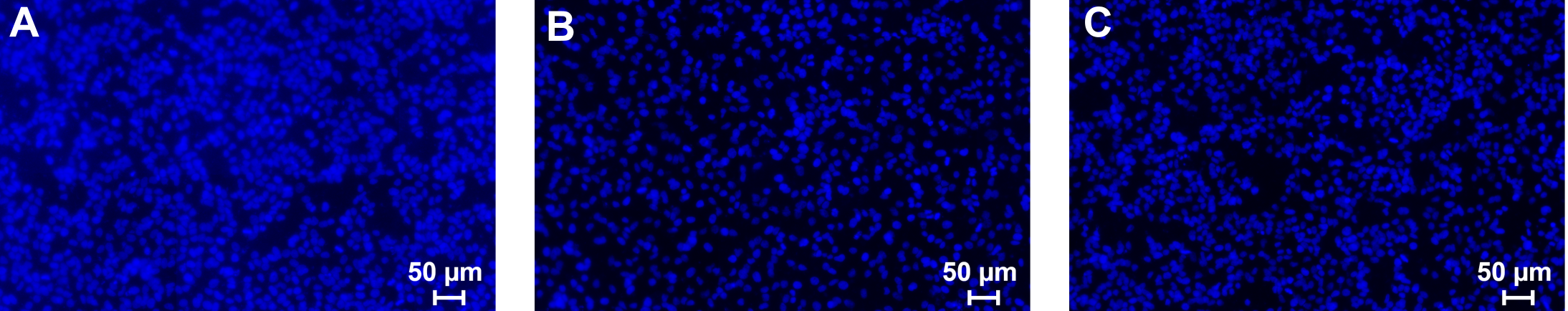

Supplement: S2 Fig — To compare the cell nuclei distribution between the three γ-H2AX fluorescence microscopy images shown in the main manuscript, nuclear DNA was visualized via DAPI staining in the corresponding cell areas receiving (A) microbeam irradiation and (B) homogeneous irradiation with a mean dose of 2 Gy and (C) no irradiation. Equal acquisition, contrast, and scaling settings were applied. Slight differences in sharpness can be due to the mounting process of the Mylar foil with the cover slide. The dense cell distribution in (A) enables easier visualization of the grating structure in the γ-H2AX channel. Even though the distribution of the cells is more even in the homogeneous case compared to the sham (cf. (B) and (C), respectively), the increased brightness seen in the γ-H2AX channel for homogeneous irradiation is not related to a denser cell distribution. (TIF) [file pone.0186005.s002.tif]
