# Supplementary material for: Increased cell survival and cytogenetic integrity by spatial dose redistribution at a compact synchrotron X-ray source
Source: PLoS One. 2017 Oct 19;12(10):e0186005. doi: 10.1371/journal.pone.0186005 (PMC5648152; doi:10.1371/journal.pone.0186005)
Supplement: S1 Appendix — (PDF) [file pone.0186005.s003.pdf]

S1 Appendix. Radiochromic film verification.

The delivered dose - controlled by the photon counting method - was verified via radiochromic films (Gafchromic EBT3, Ashland) placed directly behind the cell holder. Before the cell irradiations, we measured the absorption of the EBT3 film to correct the photon flux measured by the photon counting detector during irradiation. Films were calibrated with the Gulmay RS225A irradiation device (RS225A, xstrahl) using a tube voltage of 30 kVp and a calibrated soft X-ray ionization chamber (Type 23342, PTW). We fitted a rational function to the red channel data following Ashland (www.filmqapro.com) to achieve a calibration curve. All EBT3 films irradiated with a homogeneous field were read out with a transmission scanner (Epson Perfection V700 Photo, Epson) >48 h post-irradiation to ensure full film development.

Compared to the dose calculated with the photon counting method, the EBT3 films showed a 12-20% higher dose. Such a deviation can be caused by different systematic errors, such as the calibration of the X-ray film (energy-dependence of the film), film uniformity, scan accuracy, and fitting. In summary, we expect variations of ±12% for the EBT3 film evaluation. The errors from the photon counting method (see main manuscript) account for a dose uncertainty of ±10%. Statistical errors due to manual timing of the X-ray shutter influence both the EBT film measurement and the dose calculation. Due to the limited resolution of the scanner, MRT doses could only be evaluated with the photon counting method. Consequently, in this manuscript we show only dose values obtained via Eq (1) (cf. Materials and methods). Hence, systematic dose errors cancel themselves out when comparing homogeneous irradiation effects with microbeam irradiation effects.
