# Supplementary material for: Increased cell survival and cytogenetic integrity by spatial dose redistribution at a compact synchrotron X-ray source
Source: PLoS One. 2017 Oct 19;12(10):e0186005. doi: 10.1371/journal.pone.0186005 (PMC5648152; doi:10.1371/journal.pone.0186005)
Supplement: S1 Table — Frequency of dicentrics or centric rings per analyzed cell and their intercellular distribution in AL cells after homogeneous and microbeam irradiation with 25 keV X-rays in three experiments (Exp. I, II, III). Three replicates were performed with each irradiation condition. (PDF) [file pone.0186005.s004.pdf]

S1 Table. Detailed data on chromosome aberrations.

Exp.	X-ray dose [Gy]	Radiation	Cells scored	Dicentrics per cell ± SEM	Intercellular distribution of dicentrics						Centric rings per cell ± SEM	Intercellular distribution of centric rings				
					0	1	2	3	σ^2/μ	u-value		0	1	2	σ^2/μ	u-value
[23]	0.0	backgr. freq.	3000	0.0007	2998	2	-	-	1.0	-0.02	0	-	-	-	-	-
I	0.0	sham	540	0	540	-	-	-	-	-	0	-	-	-	-	-
	1.0	homog.	337	0.059 ± 0.014	318	18	1	-	1.25	0.54	0.047 ± 0.012	322	14	1	1.08	1.04
		microb.	294	0.014 ± 0.010	292	-	2	-	1.99	13.79	0.007 ± 0.005	292	2	-	0.99	-0.12
	2.0	homog.	340	0.124 ± 0.018	299	40	1	-	0.92	-1	0.097 ± 0.017	308	31	1	0.96	-0.48
		microb.	281	0.021 ± 0.009	275	6	-	-	0.98	-0.28	0.011 ± 0.006	278	3	-	0.99	-0.15
II	0.0	sham	603	0	603	-	-	-	-	-	0	-	-	-	-	-
	1.0	homog.	332	0.093 ± 0.019	305	24	2	1	1.23	3	0.054 ± 0.014	316	14	2	1.17	2.22
		microb.	428	0.016 ± 0.006	421	7	-	-	0.98	-0.26	0.007 ± 0.004	425	3	-	0.99	-0.13
III	0.0	sham	559	0	559	-	-	-	-	-	0	-	-	-	-	-
	1.8	homog.	278	0.144 ± 0.024	242	33	2	1	1.11	1.26	0.086 ± 0.018	255	22	1	1	-0.04
		microb.	284	0.035 ± 0.011	274	10	-	-	0.96	-0.44	0.025 ± 0.009	277	7	-	0.98	-0.32

Frequency of dicentrics or centric rings per analyzed cell and their intercellular distribution in AL cells after homogeneous and microbeam irradiation with 25 keV X-rays in three experiments (Exp. I, II, III). Three replicates were performed with each irradiation condition.

Note: σ^2/μ, dispersion ratio; u-value, test quantity (cf. [26]).

Abbreviations: backgr. freq. = background frequency, homog. = homogeneous, microb. = microbeam.
